# Nanocomposites Based on Thermoplastic Acrylic Resin with the Addition of Chemically Modified Multi-Walled Carbon Nanotubes

**DOI:** 10.3390/polym16030422

**Published:** 2024-02-02

**Authors:** Szymon Demski, Dariusz Brząkalski, Maciej Gubernat, Kamil Dydek, Paweł Czaja, Konrad Żochowski, Paulina Kozera, Zuzanna Krawczyk, Bogna Sztorch, Robert Edward Przekop, Michał Marczak, Hermann Ehrlich, Anna Boczkowska

**Affiliations:** 1 Faculty of Materials Science and Engineering, Warsaw University of Technology, 141 Wołoska St., 02-507 Warsaw, Poland; szymon.demski.dokt@pw.edu.pl (S.D.); konrad.zochowski2.stud@pw.edu.pl (K.Ż.); paulina.kozera@pw.edu.pl (P.K.); anna.boczkowska@pw.edu.pl (A.B.); 2 Centre for Advanced Technologies, Adam Mickiewicz University in Poznań, 10 Uniwersytetu Poznańskiego St., 61-614 Poznań, Poland; d.brzakalski@gmail.com (D.B.); bogna.sztorch@gmail.com (B.S.); r.przekop@gmail.com (R.E.P.); hermann.ehrlich@amu.edu.pl (H.E.); 3 Faculty of Materials Science and Ceramics, AGH University of Science and Technology, Al. Mickiewicza 30, 30-059 Cracow, Poland; maciej.gubernat@agh.edu.pl; 4 The Aleksander Krupkowski Institute of Metallurgy and Materials Science, Polish Academy of Sciences, 25 Reymonta St., 30-059 Kraków, Poland; p.czaja@imim.pl; 5 Institute of Materials, Ecole Polytechnique Fédérale de Lausanne, Station 12, CH-1015 Lausanne, Switzerland; zuzanna.krawczyk@epfl.ch; 6 Faculty of Mechanical and Industrial Engineering, Warsaw University of Technology, 85 Narbutta St., 02-524 Warsaw, Poland; michal.marczak@pw.edu.pl

**Keywords:** carbon nanotubes, nanocomposites, acrylic resin, electrical properties, mechanical properties, chemical modification

## Abstract

The main goal of this work was an improvement in the mechanical and electrical properties of acrylic resin-based nanocomposites filled with chemically modified carbon nanotubes. For this purpose, the surface functionalization of multi-walled carbon nanotubes (MWCNTs) was carried out by means of aryl groups grafting via the diazotization reaction with selected aniline derivatives, and then nanocomposites based on ELIUM^®^ resin were fabricated. FT-IR analysis confirmed the effectiveness of the carried-out chemical surface modification of MWCNTs as new bands on FT-IR spectra appeared in the measurements. TEM observations showed that carbon nanotube fragmentation did not occur during the modifications. According to the results from Raman spectroscopy, the least defective carbon nanotube structure was obtained for aniline modification. Transmission light microscopy analysis showed that the neat MWCNTs agglomerate strongly, while the proposed modifications improved their dispersion significantly. Viscosity tests confirmed, that as the nanofiller concentration increases, the viscosity of the mixture increases. The mixture with the highest dispersion of nanoparticles exhibited the most viscous behaviour. Finally, an enhancement in impact resistance and electrical conductivity was obtained for nanocomposites containing modified MWCNTs.

## 1. Introduction

Polymeric materials are widely used in all sectors of the economy, and for most of the applications, the properties of neat polymers are sufficient. Nevertheless, more advanced polymers are in demand in the automotive, aviation, and electronics industries [1]. To meet the expectations of these industries, an increase in themechanical, electrical, or thermal properties of materials is required. This could be achieved through the utilization of high-performance polymers or polymer matrix composites [2,3]. However, there are numerous disadvantages of the first solution, including expensive raw materials and high requirements for equipment and processing parameters, while the latter can be achieved with the addition of metal particles and oxides (silver, copper, aluminium oxide) or carbon-based materials such as carbon nanotubes (CNTs), graphene nanoplatelets, or carbon fibres [4].

Thanks to their exceptional mechanical properties, high electrical conductivity, large aspect ratio, and low percolation threshold [5,6,7,8], carbon nanotubes, both single- (SWCNTs) and multi-walled (MWCNTs), have been extensively used as reinforcement to provide increased mechanical strength, as well as enhanced electrical and/or thermal conductivity of several polymers, including various polyolefins (high-density polyethylene, ultra-high-molecular-weight polyethylene, polypropylene), polystyrene, polyamide 6, polyamide 11, polycarbonate, polyaniline, polyimide, poly(vinyl alcohol), or epoxy resins [9,10]. Poly(methyl methacrylate) (PMMA)/CNT composites have been studied as well in terms of their mechanical, electrical, thermal or antimicrobial properties [11,12,13].

A common issue regarding the compatibility of CNTs with the polymer matrix, which strongly affects the performance of the resulting composite, is the tendency of CNTs to agglomerate. Their shape and the structure of highly conjugated unsaturated carbon–carbon bonds result in strong intermolecular π-π interactions (π-stacking), which in turn cause strong agglomeration, solvophobic interactions, and poor dispersion in several media, including polymers [14]. There are several physical methods utilized to facilitate the deagglomeration process, such as sonication in solutions or shear mixing in polymer melts; however, the addition of surfactants or other surface-active agents is often required to stabilize the nanotubes [15,16], as the process is thermodynamically reversible [17]. In order to mitigate this issue, multiple approaches have been applied to reduce the contribution of attractive forces between CNTs in favour of interaction with the medium they are dispersed within [18]. The available methods utilize either physical adsorption of molecules of dispersing agents (surfactants) onto the CNT wall, or chemical modification of the nanotube itself, which can be carried out by several techniques, including halogenation, esterification, amidation, etherification, salinization, radical grafting or partial oxidation, which can also be applied in conjunction with other methods as an activation/pre-treatment step. However, partial oxidation may also affect the CNT structure and its capabilities in terms of thermal or electrical conductivity. Therefore, non-oxidizing methods may be considered more attractive, especially if a one-step procedure can be performed. An example of such a procedure is diazonium surface grafting, where diazonium salts are produced and coupled with the CNT wall; the technique is proven to be effective and particularly simple to implement [19,20]. Diazonium surface grafting chemistry is well-known and has been successfully applied to a number of various materials [21,22].

In recent years, one of the rapidly developing materials has been thermoplastic acrylic resin Elium^®^, which combines some characteristics of thermoplastics and thermosets. Low viscosity, high strength and lightweight, adjustable reactivity, very good UV resistance, and the possibility of curing at room temperature are just a few of its many advantages. Moreover, as a thermoplastic polymer, it can be recycled by chemical and mechanical processes and is less toxic (styrene and bisphenol A free) than typical thermosets. Elium^®^ resin can be successfully used in the wind energy, marine, construction, and transportation sectors. In the literature, one can find studies on ELIUM resin and the analysis of its polymerization reaction [23], the effect of the initiator content [24], and the effect of moisture absorption [25], as well as mechanical behaviour [26,27]. However, research on the electrical and mechanical properties of Elium^®^/CNT nanocomposites has not yet been studied in depth.

In this paper, MWCNTs were chemically modified and then used as nanofillers in Elium^®^-based nanocomposites. The system proposed in this work has not been presented in the literature before, and the developed modification of acrylic resin could be successfully applied in many sectors, i.e., automotive, wind energy, or defence industries. The main aim of the study is to show the effect of the chemical modification of MWCNTs on the selected properties of Elium^®^/MWCNT nanocomposites. To achieve this, MWCNTs were chemically modified by diazo grafting utilizing three different aniline modifier derivatives: aniline, p-nitroaniline and anthranilic acid, and their effects on the rheological, electrical and mechanical properties of the fabricated nanocomposites were examined.

## 2. Materials and Methods

### 2.1. Materials

A novel thermoplastic acrylic resin Elium^®^188O (Arkema, Colombes, France), which has a low viscosity of 100 mPa*s and a density of 1.01 g cm^−3^, was used as the polymer matrix for the fabricated nanocomposites. MWCNTs, with the trade name NC7000 from Nanocyl (Sambreville, Belgium) and the following properties: average diameter—9.5 nm, average length—1.5 µm, and purity—90%, were used as nanofillers after chemical modification. Materials and chemicals were purchased from the following sources: potassium bromide (KBr), dimethylformamide (DMF), isoamyl nitrite, and all the aniline compounds (aniline, p-nitroaniline, anthranilic acid) were purchased from Merck (Poznań, Poland); calcium oxide (CaO), isopropanol, toluene, methylene chloride, and tetrahydrofuran (THF) were purchased from Avantor Performance Materials (Gliwice, Poland). DMF was dried and purified by distilling it from CaO and was kept over 3A molecular sieves. KBr was dried at 105 °C overnight and kept in an airtight glass container. The other reagents were used as received.

#### 2.1.1. Surface Modification of MWCNTs

The surface functionalization of MWCNTs was carried out by means of aryl groups grafting via the diazotization reaction of the selected aniline derivatives with isoamyl nitrite, under nonaqueous conditions, based on the methodology as described in the literature [19,20]. The procedure was as follows:

(1) The dispersion 1 g of MWCNTs for 1 h in 400 mL of dry (DMF) at 50 °C in a two-necked round-bottom flask, carried out in an ultrasonic bath; (2) the addition of a solution of 100 mmol of the selected aniline derivative in 200 mL DMF to the MWCNTs suspension; (3) placement of the solution in a reaction setup equipped with a reflux condenser and a magnetic stirrer and stabilization of the temperature at 60 °C; (4) the dropwise addition of isoamyl nitrite over a time of 1 h; (5) heating of the system for 20 h; (6) the addition of 2 mL of anhydrous acetic acid in 20 mL of DMF and an increase in the temperature to 100 °C to remove any possible unreacted residues of diazonium salts; (7) the hot filtering of carbon nanotubes through filter paper; (8) thorough washing of the nanotubes successively with DMF, isopropanol, toluene, methylene chloride, and THF and again with isopropanol in order to remove excess unreacted modifier and its decomposition products; (9) a final washing of the nanotubes with isopropanol, performed in an ultrasonic bath; (10) drying the modified nanotubes in a drying cabinet for 24 h at 40 °C; and (11) grinding the modified nanotubes with a pestle and mortar until a fine powder was obtained.

**Figure 1:**
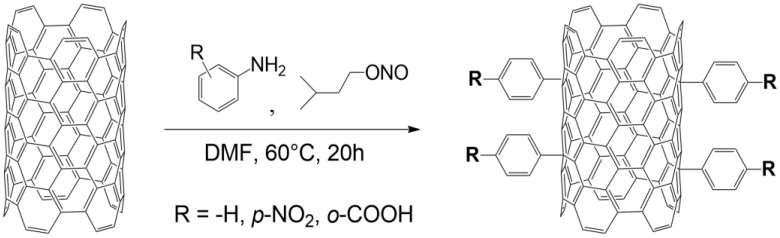
Reaction scheme for grafting MWCNTs with the selected aniline derivatives as performed in this work.

The scheme of the grafting reaction is shown in Figure 1, and Table 1 presents the modifications used.

#### 2.1.2. Preparation of Acrylic Resin/MWCNT Mixtures

Elium^®^/MWCNTs mixtures were prepared by mixing resin with the following MWCNTs concentrations: 0.02 wt%, 0.10 wt%, and 0.15 wt%. For each MWCNT concentration, four dispersions were prepared. One was prepared with neat MWCNTs and three were prepared with MWCNTs after surface modifications: MWCNT-AN, MWCNT-NAN, and MWCNT-ABA. After mechanical mixing, Elium^®^/MWCNT dispersions were treated with ultrasonic waves, which is a commonly used method in the case of CNTs [28,29]. The ultrasonication was performed using a VCX1500 (Sonics & Materials, Newtown, CT, USA) ultrasonic processor with a maximum frequency of 20 kH for 1 h at an amplitude of 40%. Ultrasonic waves were on for 9 s out of every 14 s of the cycle to prevent the temperature from increasing over 45 °C. After ultrasonication, 2.0 wt% of the initiator (Dibenzoyl peroxide, Acros Organics, Antwerp, Belgium) was added to the prepared mixtures, which were then placed in a mould for 24 h at room temperature. Finally, manufactured composites were post-cured at 80 °C for 24 h.

### 2.2. Methods

Fourier Transform Infrared (FT-IR) spectra were recorded utilizing a Nicolet iS 50 Fourier transform spectrophotometer (Thermo Fisher Scientific, Waltham, MA, USA) equipped with a diamond ATR unit with a resolution of 0.09 cm^−1^. Standard KBr pellets were made using 0.1 wt% of the sample. For the best results, the chosen MWCNTs were first ground with KBr at 1.0 wt% loading and the obtained homogeneous, dark-grey powder was then diluted to 0.1 wt.% by grinding with a fresh portion of pure KBr.

Raman measurements were performed using a Horriba LabRam HR spectrometer. The 532 nm diode laser, 1800 grating, and 100× objective were used during the measurements. Each sample was tested 5 times. Each accumulation lasted for 7 min, and the power was adjusted to approx. 0.3 mW, to prevent sample degradation. The deconvolution of the primary bands on the obtained Raman spectra was carried out using Fityk 1.3.1 software and the Voigt function [30].

Transmission Electron Microscopy (TEM) analysis was conducted with the use of a Titan Themis X-FEG G3 Cs-corrected S/TEM microscope (Thermo Fisher Scientific, Waltham, MA, USA). In order to perform the observations, suspensions of carbon nanotubes in 15 mL of ethyl alcohol were prepared, which were then dispersed for 5 min with 40% amplitude using a 130 W ultrasonic processor from Sonics. The prepared suspensions were then applied to the mesh substrate and introduced into the microscope chamber. ImageJ 1.53t was used to measure the diameters of the carbon nanotubes in TEM images [31]. For each sample, 50 measurements were made, and then the obtained values were averaged. The Gatan Digital Micrograph programme was used to determine interplanar distances in carbon nanotubes in high-resolution TEM (HRTEM) images.

The macrodispersion state of MWCNTs was characterized by transmission light microscopy. Samples in the form of an uncured mixture of resin and carbon nanotubes were used for observations. First, the samples were deposited on the surface of a microscope glass and then observed through a PZO Biolar transmission microscope (Biolar, Warsaw, Poland).

The viscosity measurements of the prepared resin mixtures with modified MWCNTs were conducted using a DV-II + Pro Viscometer (Brookfield, Canada). The tests were carried out at 25 °C from 1 to 200 rpm, using 8 mL of the mixtures after the sonification process. The used spindle, model SC4-21, had a fixed shear rate value of 0.93 N per 1 rpm.

Impact resistance testing is used to study the behaviour of materials under dynamic loads. In this paper, the influence of the addition of chemically modified MWCNTs on the impact resistance of nanocomposites was investigated in accordance with PN-EN ISO 179-1 standard [32]. The test was carried out using a Charpy Resil 5.5 CEAST hammer with a 4 J impact energy.

A Keithley 6221/2182A (Tektronix, Beaverton, OR, USA) measurement set was used to study the effect of the chemical modification of MWCNTs on the electrical conductivity of fabricated MWCNT-based nanocomposites. The tests were carried out using the four-point method, and the measurement device was equipped with a test cell with copper electrodes. Four samples (dimensions 10 × 10 mm) collected from different areas were tested for each nanocomposite. The range of current was 0.5–100 nA, and the test itself was conducted at room temperature. To ensure better contact between the sample and the electrode, a thin layer of highly conductive silver paste was applied.

## 3. Results

### 3.1. FT-IR Spectroscopy

**Figure 2:**
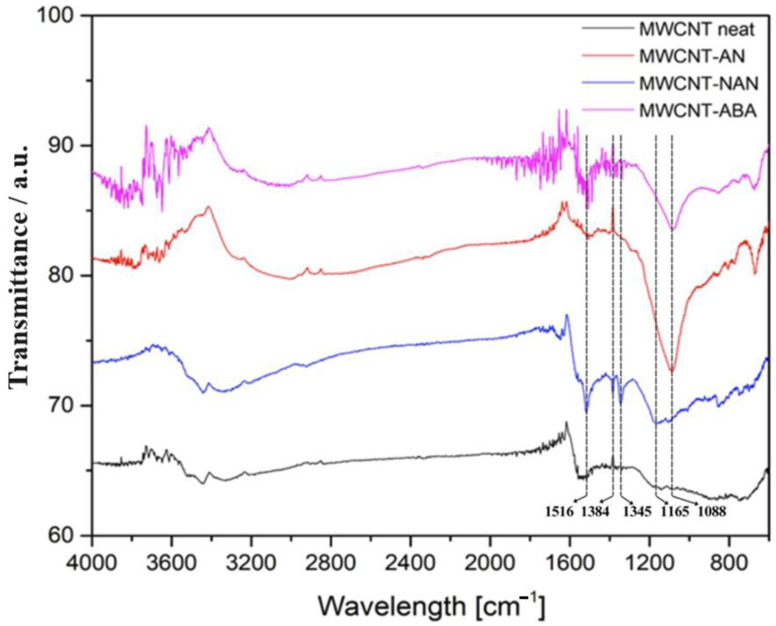
FT-IR spectrum of the MWCNTs treated by aryl grafting with aniline derivatives compared to the neat MWCNTs.

In order to confirm the effectiveness of the chemical surface modification of MWCNTs, FT-IR spectra of the fabricated materials were obtained and compared with the spectra of the neat MWCNTs (Figure 2). FT-IR analysis enabled the observation of functional groups grafted onto the surface of the MWCNTs as a result of the diazo treatment with the selected aniline derivatives. For the MWCNTs treated with aniline (MWCNT-AN), a new strong band with a maximum at 1088 cm^−1^ is visible. This band corresponds to the in-plane bending of the C-H bonds of aromatic rings [33] and is absent in the spectrum collected for the neat carbon nanotubes. For the MWCNT-NAN system, that is, the MWCNTs treated with p-nitroaniline, a new intense band with a maximum at 1165 cm^−1^ can be seen, which, similar to MWCNT-AN, is in the aromatic C-H in-plane bending range. In addition, bands at 1516, 1384, and 1345 cm^−1^ are visible, corresponding to the stretching of the nitro group, which confirms the presence of the nitroaryl group on the MWCNT surface. For the MWCNT-ABA system, which was treated with o-aminobenzoic (anthranilic) acid, no carboxylic groups were observed on the FT-IR spectrum (absence of characteristic C=O stretching band), which is virtually identical to that of the MWCNT-AN system.

**Figure 3:**
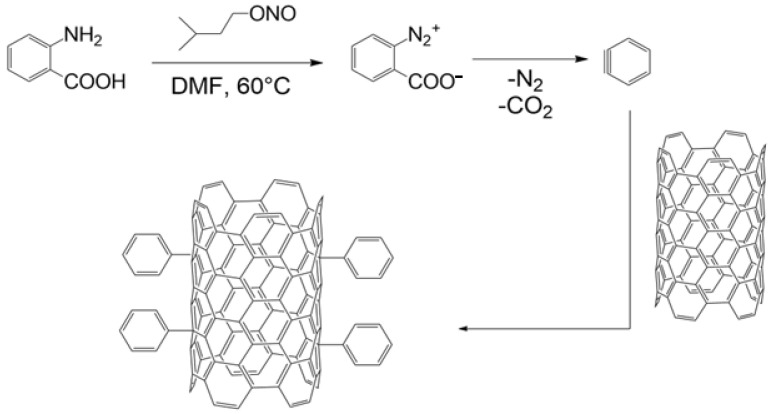
Reaction pathway of anthranilic acid deazotization–decarboxylation leading to benzyne formation and the grafting thereof on the surface of MWCNTs.

This confirms that under the given reaction conditions, the diazonium salt of anthranilic acid undergoes a dediazotization–decarboxylation reaction to benzyne, which is the active species to react with MWCNTs, therefore giving the product of phenyl group grafting instead of the o-carboxyphenyl one [34] (Figure 3). It is worth noting that such an effect may be beneficial, as it allows for a rather selective introduction of a phenyl group onto the surface of MWCNTs (and probably other related carbon nanomaterials, such as graphene) with the use of anthranilic acid, which, as opposed to aniline, is a non-toxic, shelf-stable, well-known metabolite in human, animals, and plants [35].

### 3.2. Transmission Electron Microscopy

**Figure 4:**
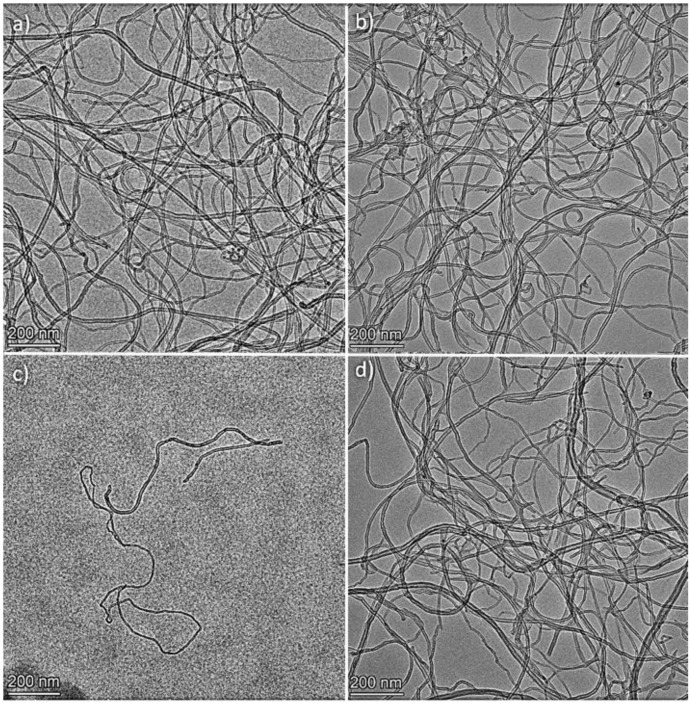
TEM images of (**a**) MWCNT neat, (**b**) MWCNT-AN, (**c**) MWCNT-NAN, and (**d**) MWCNT-ABA.

TEM observations allowed us to compare the morphology of the MWCNTs and the structural changes that occurred before and after their modification. TEM showed that carbon nanotubes were not fragmented during the modification, which is important for maintaining a high aspect ratio, thanks to which they are effective in enhancing the mechanical, electrical, and thermal properties of polymer matrices (Figure 4). The length of the nanotubes in all cases exceeded 1 μm. The measured diameter for neat MWCNT was 12.4 nm (SD = 4.4 nm), and it did not differ much for the material after modification, which was, respectively, MWCNT-AN—12.5 nm (SD = 3.5 nm), MWCNT-NAN—15.2 nm (SD = 4.2 nm), and MWCNT-ABA—12.1 nm (SD = 4.5 nm). The modification did not cause excessive twisting, tangling, or sticking of the nanotubes. In the samples with modified nanotubes, there were only a few traces of the process noticeable in the form of regions of glued nanotubes, which were especially visible in the case of MWCNT-AN (Figure 4b).

**Figure 5:**
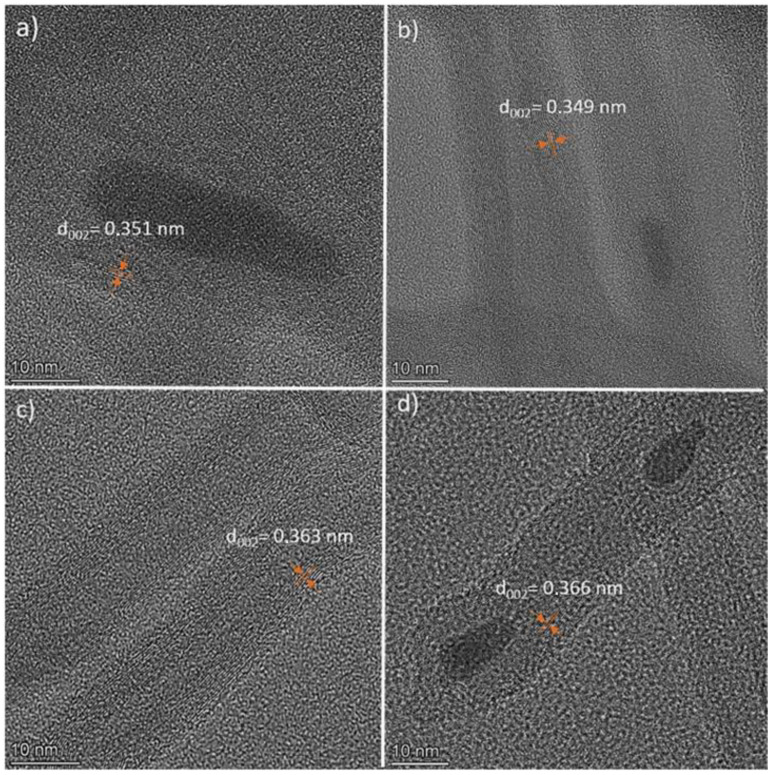
High-resolution TEM images of (**a**) MWCNT neat, (**b**) MWCNT-AN, (**c**) MWCNT-NAN, and (**d**) MWCNT-ABA.

The influence of the modifications on the structure of the nanotubes was analysed. Interplanar distances d002 measured based on the HRTEM images allowed us to determine the probability of deterioration for the structure of the nanotubes modified with p-nitroaniline and anthranilic acid. d002 for neat MWCNTs was 0.351 nm, while for MWCNT-NAN it was 0.363 nm, and for MWCNT-ABA, it was 0.366 nm (Figure 5). The increase in the value of the d002 parameter indicates a regional disturbance of the structure of the nanotubes, which, in this case, could have been caused by their partial oxidation under the influence of oxidative modifying agents. Significant changes in d002 were not observed in the case of MWCNT-AN. The nanoparticles visible in the nanotube core are the remains of the nanotube growth catalyst.

### 3.3. Raman Spectroscopy

**Figure 6:**
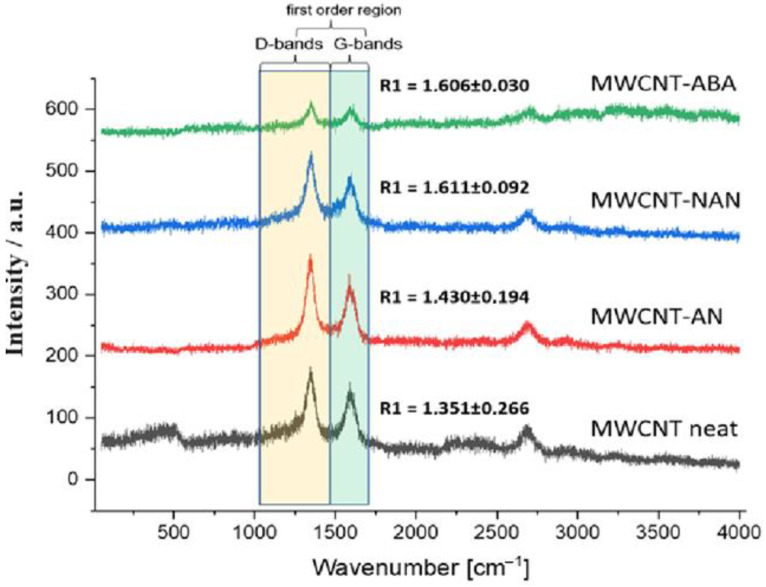
Raman spectra of MWCNT neat, MWCNT-AN, MWCNT-NAN, and MWCNT-ABA.

To confirm the observed local structural changes in the HRTEM images (Figure 5), a Raman spectroscopic analysis of the nanotubes before and after the modification was performed. The analysis of the primary bands and the comparison of the values of the R1 parameter, which is the quotient of the intensity of the D band and the G band, allowed us to confirm that the observed changes are also traceable using Raman spectra (Figure 6). The presence of the D band in the spectrum at the position of about 1350 cm^−1^ is associated with the presence of defects in the nanotube structure and the consequent laser scattering. The G-band centred at about 1580 cm^−1^ is the primary band for the ordered graphite structure. The ID/IG = R1 quotient is the basic parameter for comparing the degree of disorder in sp2 materials, including carbon nanotubes [36]. R1 for neat MWCNT was 1.351 (SD = 0.266), and it was the lowest one out of the tested samples. This proves the deterioration of the structural order of the nanotubes after their modification, the largest of which was for the MWCNT-NAN and MWCNT-ABA samples, for which the R1 values were 1.611 (SD = 0.092) and 1.606 (SD = 0.030), respectively. From the perspective of ensuring the maintenance of the lack of disturbance of the primary structure of nanotubes, aniline turned out to be the most appropriate modifier.

### 3.4. Microscopy Observations

**Figure 7:**
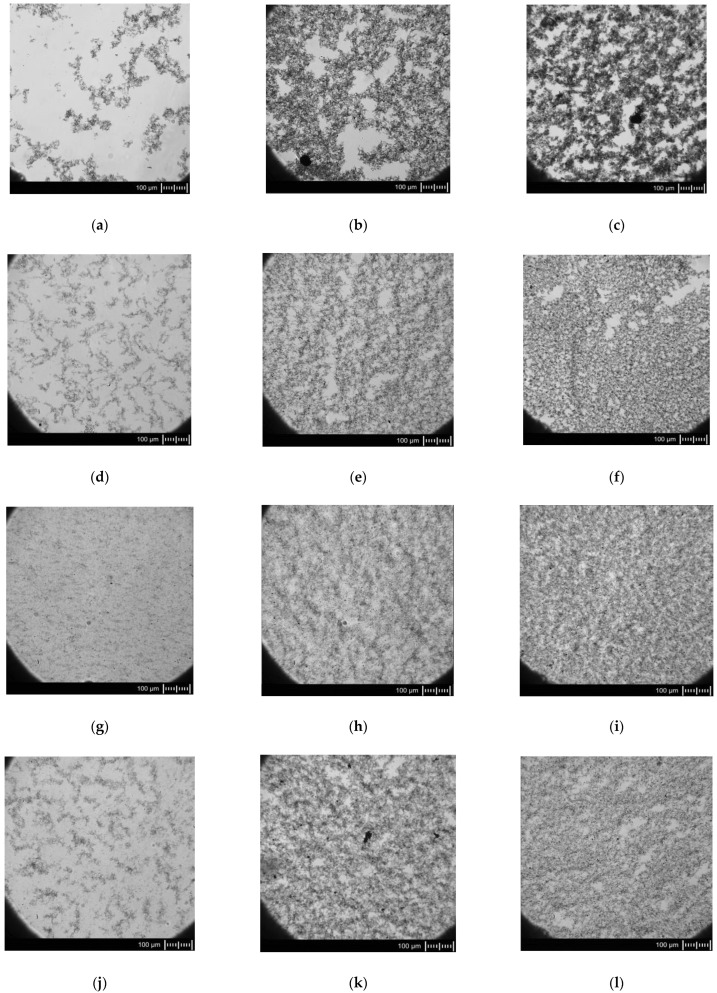
Microscope observations of he prepared mixtures, (**a**) ELIUM + 0.02 wt%MWCNT neat, (**b**) ELIUM + 0.10 wt%MWCNT neat, (**c**) ELIUM + 0.15 wt%MWCNT neat, (**d**) ELIUM + 0.02 wt%MWCNT-AN, (**e**) ELIUM + 0.10 wt%MWCNT-AN, (**f**) ELIUM + 0.15 wt%MWCNT-AN, (**g**) ELIUM + 0.02 wt%MWCNT-NAN, (**h**) ELIUM + 0.10 wt%MWCNT-NAN, (**i**) ELIUM + 0.15 wt%MWCN(T-NAN, (**j**) ELIUM + 0.02 wt%MWCNT-ABA, (**k**) ELIUM + 0.10 wt%MWCNT-ABA, and (**l**) ELIUM + 0.15 wt%MWCNT-ABA.

Morphological analysis is very important for the evaluation of the dispersion state of carbon nanotubes in the polymer matrix. The dispersion of both unmodified and functionalized MWCNTs in an acrylic resin matrix was assessed regarding the agglomerate distribution (so-called macrodispersion) using a light optical microscope PZO Biolar (Biolar, Warsaw, Poland), and the results are shown in Figure 7. When introduced into the acrylic matrix, the neat MWCNTs agglomerated strongly. It could be seen that in the case of ELIUM/MWCNT neat mixtures (shown in Figure 7a–c), several isolated large agglomerates of MWCNTs were present as well as many voids with only ELIUM resin; this is an effect which has been already described in the literature [13,37]. The presence of large MWCNT agglomerates indicates that the entangled MWCNTs have not been separated. This behaviour of the material proves that the intrinsic van der Waals attractions among carbon nanotubes were stronger than the poor interfacial interaction between MWCNTs and the PMMA matrix [38]. It should be also noted that all of the surface functionalization methods proved to be effective in reducing agglomerate formation and controlling the dispersibility of the nanotubes. Generally, the chemically modified MWCNTs were better dispersed throughout the acrylic matrix than the neat MWCNTs. For the modified samples, the homogeneous filler dispersion and no obvious large agglomerates were observed, as shown in Figure 7d–l. After the chemical treatment, the van der Waals forces among carbon nanotubes were weakened. Therefore, the entanglement of MWCNTs became loose, forming smaller agglomerates [38]. Moreover, better dispersion means more individual MWCNTs that form a continuous network of connections in the polymer matrix. Similar results were reported for –OH functionalization of MWCNTs [39]. Analogously, as discussed in the FT-IR section, MWCNT-AN and MWCNT-ABA showed very similar behaviour, exhibiting comparable levels of dispersion caused by the same chemical surface character, while the MWCNT-NAN system was dispersed much more effectively. This outcome is due to the highly polar character of the nitro group, which provides stronger interactions with the methacrylate monomers and oligomers. The above findings demonstrate the effectiveness of the selected approach in achieving good dispersion of MWCNTs in the methacrylic matrix.

### 3.5. Viscosity

**Figure 8:**
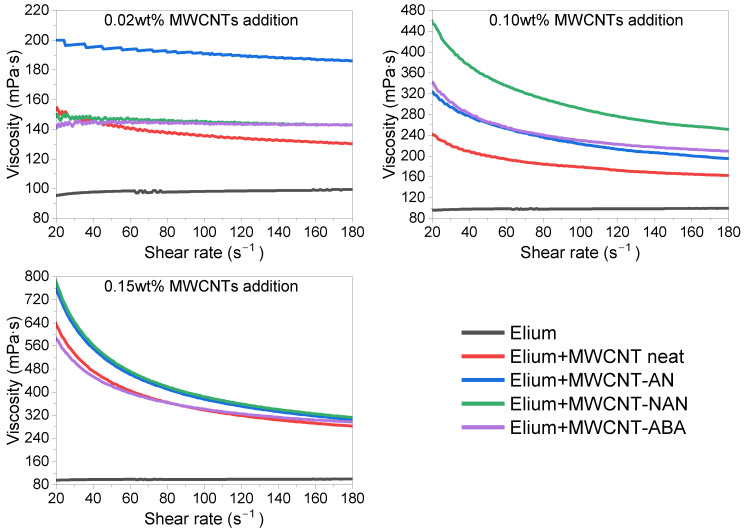
Viscosity results.

MWCNTs are known as nanofillers that significantly change the rheological properties of various thermoplastics and thermosets, mainly due to their high aspect ratio [40]. The effects of the chemical surface modification and the content of MWCNTs on the viscosity of Elium^®^/MWCNTs mixtures were examined, and the results are shown in Figure 8. The Elium^®^188O resin used, without MWCNTs, was characterized by a viscosity of 100 mPa·s and Newtonian fluid-like behaviour [41]. There is no doubt that the addition of MWCNTs significantly affects the rheological behaviour of Elium/MWCNT mixtures, causing them to act as shear-thinning (non-Newtonian) fluids [42]. Analysing the effect of surface functionalization of MWCNTs, it could be noted that the modification with aniline derivatives affected the increase in viscosity of the mixtures, especially at lower concentrations. The functional groups provided by chemical modification prevented the agglomeration of MWCNTs, resulting in better dispersion of the nanofiller in the polymer matrix and increasing the surface area of contact between the resin and filler, which, consequently, increased viscosity as compared with the neat MWCNTs [43]. It is well-known in the literature [44] that as MWCNTs increase in a mixture, their viscosity increases, which was also observed for the mixtures analysed in this work. The highest viscosity of about 300 mPa ·s was obtained for an MWCNT concentration of 0.15 wt% at a shear rate of 180 s^−1^, which is noteworthy because modified resin with such characteristics may be successfully used in several techniques for manufacturing fibre-reinforced polymers [45].

### 3.6. Impact Resistance

**Figure 9:**
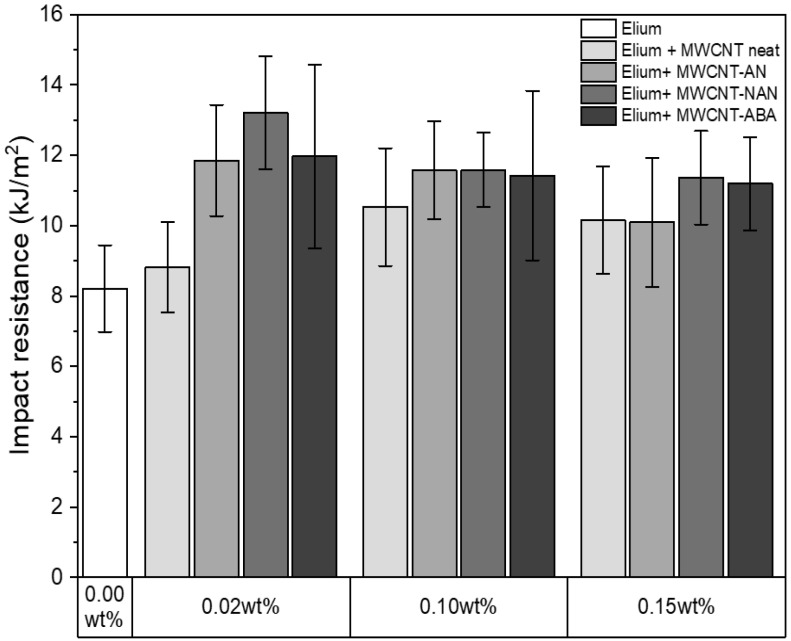
Impact resistance of the fabricated nanocomposites.

Impact resistance tests were conducted to compare the energy absorption capacity of samples filled with different concentrations of neat and modified MWCNTs, and the results are demonstrated in Figure 9. The lowest impact resistance value of 8.2 kJ m^−2^ (SD = 1.23 kJ m^−2^) was obtained for neat Elium^®^ resin, while an increase in dynamic load resistance was observed for all the nanocomposites filled with modified MWCNTs. This mechanical behaviour is in agreement with the well-known elastic properties of MWCNTs, which are capable of withstanding tensional, torsional, and compression stresses [46]. MWCNTs are also characterized by high surface energy, whereby microcrack bridging can be observed [47]. It is noteworthy that the highest impact strength values were obtained for the chemically modified MWCNTs with 0.02 wt%, where the Elium with 0.02 wt% MWCNT-NAN system showed an improvement of 61% and 50% compared to neat resin and Elium with unmodified MWCNTs, respectively. Such results could be explained by the best dispersion of MWCNTs in the polymer matrix for this modified nanocomposite, as presented in Figure 7g. Moreover, it was observed that with an increase in the concentration of modified MWCNTs with any of the proposed compounds, there was a decrease in the resistance to dynamic loads, which was probably related to the entanglement phenomena of nanoparticles, resulting in the deterioration of the MWCNT–resin interface and the appearance of defects in the structure of the nanocomposite [48]. This phenomenon was also observed in another work and may indicate that for the developed MWCNTs modifications, the inclusion threshold occurs at filler concentrations below 0.10 wt% [49].

### 3.7. Electrical Conductivity

**Figure 10:**
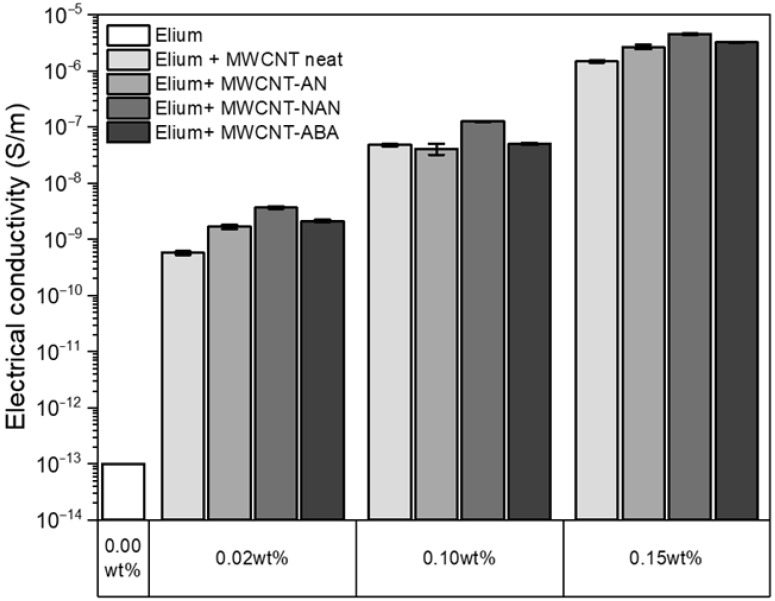
Electrical conductivity of the fabricated nanocomposites.

To evaluate the effectiveness of the surface functionalization of the MWCNTs, the electrical conductivity of the fabricated nanocomposites was examined, and the results are presented in Figure 10. The electrical conductivity value for neat Elium^®^ resin was assumed to be 10–13 S m^−1^, which corresponds to that of neat PMMA [50]. It was observed that with the addition of MWCNTs of a concentration of 0.02 wt%, the electrical conductivity of the nanocomposites increased by four orders of magnitude. The highest value for this concentration was obtained for the Elium + MWCNT-NAN system, and the lowest was obtained for Elium + non-modified MWCNTs. The values were 3.7 × 10^−9^ S m^−1^ and 5.7 × 10^−10^ S m^−1^, respectively. The higher values obtained for the modified MWCNTs can be attributed to the better dispersion of nanoparticles in the polymer matrix than for unmodified MWCNTs, resulting in the formation of more conductive pathways between the conductive filler particles [51]. It is well-known that as the concentration of MWCNTs increases, the electrical conductivity of the nanocomposites increases as well, and the same phenomenon was observed for the fabricated materials elsewhere [52]. The highest value (4.57 × 10^−6^ S m^−1^) was obtained for the Elium + 0.15 wt% MWCNT-NAN nanocomposite, which represented an improvement in electrical conductivity of almost seven orders of magnitude as compared to neat Elium^®^ resin. In general, the highest values were obtained for the MWCNT-NAN system, which can be explained by the better degree of dispersion that was obtained in the acrylic matrix. However, the differences between the values for the developed MWCNTs modifications were negligible.

## 4. Conclusions

In this work, the effect of chemical surface modifications of MWCNTs on the electrical and mechanical properties of ELIUM^®^ thermoplastic acrylic resin-based nanocomposites was investigated. The surface functionalization of MWCNTs was carried out by means of aryl groups grafting via the diazotization reaction of selected aniline derivatives, and then MWCNTs were dispersed in an acrylic resin by ultrasonication. The presence of the new bands on the FT-IR spectra, i.e., at 1088–1165 and 1516, 1384 and 1345 cm^−1^, corresponding, respectively, to the bending of the C-H bonds of aromatic rings in the plane and the stretching of the nitro group confirmed the effectiveness of the implemented chemical modification of MWCNTs. It was found that the performed surface modifications provided a better dispersion of MWCNTs in the acrylic resin, which was verified by microscopic observations. In addition, the conducted TEM analysis showed that the modification did not cause excessive twisting, tangling, or sticking of the nanotubes. However, for the MWCNT-NAN and MWCNT-ABA modifications, the interplanar distances d002 increased. Raman spectroscopy showed that the MWCNT-NAN and MWCNT-ABA modifications were characterized by a higher R1 parameter when compared with pure MWCNTs, indicating a higher degree of disorder in the sp2 materials. The viscosity tests confirmed that with an increasing concentration and better dispersion of MWCNTs, the viscosity of the mixture increases. Comparing the results, the addition of 0.15 wt% MWCNT-NAN increased the viscosity to a value of 320 mPa*s, whereas that of the reference sample was 100 mPa*s. It was noted that the best dispersion of MWCNTs was observed for the -NAN modification, which led to obtaining the highest value of impact resistance and electrical conductivity for the Elium/MWCNT nanocomposites. For the NAN modification with a concentration of 0.02 wt%, the improvement in impact resistance was 61% and 50%, as compared with pure resin and neat MWCNTs, respectively. Moreover, the study of electrical properties allowed us to conclude, that as the concentration of MWCNTs increased, the electrical conductivity of the nanocomposites increased, and that the performed modifications slightly enhanced its value when compared to neat MWCNTs. However, the influence of the type of aniline derivative used was negligible.

## Figures and Tables

**Table 1:** The aniline modifications used in this work.

Aniline Modifier Derivative	Aniline Modifier Structure	MWCNTs System Abbreviation
None	-	MWCNT neat
Aniline	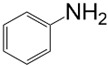	MWCNT-AN
*p*-nitroaniline	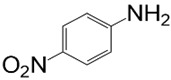	MWCNT-NAN
anthranilic acid (*o*-aminobenzoic acid)	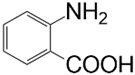	MWCNT-ABA

## Data Availability

All data are available in the main text.
